# The Milk Microbiota of the Spanish Churra Sheep Breed: New Insights into the Complexity of the Milk Microbiome of Dairy Species

**DOI:** 10.3390/ani10091463

**Published:** 2020-08-20

**Authors:** Cristina Esteban-Blanco, Beatriz Gutiérrez-Gil, Héctor Marina, Rocío Pelayo, Aroa Suárez-Vega, Alberto Acedo, Juan-José Arranz

**Affiliations:** 1 Departamento de Producción Animal, Facultad de Veterinaria, Universidad de León, Campus de Vegazana s/n, 24071 León, Spain; cestb@unileon.es (C.E.-B.); bgutg@unileon.es (B.G.-G.); hmarg@unileon.es (H.M.); rpelg@unileon.es (R.P.); asuav@unileon.es (A.S.-V.); 2 Biome Makers Inc., West Sacramento, CA 95605, USA; acedo@biomemakers.com

**Keywords:** Assaf, Churra, dairy sheep, milk microbiota, 16S rRNA gene sequencing

## Abstract

**Simple Summary:**

In the last decade, the number of studies focused on the study of the microbiota of different tissues, organs, or physiological fluids has considerably increased. The milk of dairy species is an important and continuous source of commensal, mutualistic and potentially probiotic bacteria. Second-generation sequencing technologies have been applied to characterise the milk microbiota of dairy cows, whereas the study of the sheep milk microbiota is scarce. In the present study, we aimed to explore the bacterial diversity and composition of milk samples from the Churra sheep breed, a rustic autochthonous breed from the region of Castilla y León (Spain). Moreover, this study tries to clarify the complex bacterial composition of sheep milk comparing the results presented here with previous research on the milk microbiota of the Assaf sheep breed. This assessment has shown that the milk microbiota of ewes from one flock of the Assaf breed is more diverse than the milk microbiota reported here for two different flocks of Churra sheep. The study also provides a step into a better understanding of the link between the bacterial milk composition in these two sheep breeds and somatic cell count, an indicator trait of subclinical mastitis resistance in dairy sheep.

**Abstract:**

Milk from healthy animals has classically been considered a sterile fluid. With the development of massively parallel sequencing and its application to the study of the microbiome of different body fluids, milk microbiota has been documented in several animal species. In this study, the main objective of this work was to access bacterial profiles of healthy milk samples using the next-generation sequencing of amplicons from the 16S rRNA gene to characterise the milk microbiome of the Churra breed. A total of 212 samples were collected from two Churra dairy farms with a different management system. The core milk microbiota in Churra ewes includes lesser genera (only two taxa: Staphylococcus and Escherichia/Shigella) than studies reported in other dairy species or even in a previous study in Assaf sheep milk. We found that diversity values in the two flocks of Churra breed were lower than the diversity of the milk microbiota in Assaf. The non-metric multidimensional scaling (NMDS) ordination using Bray-Curtis distance separates samples based on their microbiota composition. The information reported here might be used to understand the complex issue of milk microbiota composition.

## 1. Introduction

The study of the microbial communities found in milk has gained increasing interest in recent years. The classical concept of the sterility of the mammary gland and milk has been challenged by the results obtained in the last decade using bacterial DNA-based methodologies [1,2]. The recent significant development in culture-independent techniques, especially using 16S rRNA gene sequencing, has led to new studies dedicated to understanding the composition, the diversity and the biological roles of the bacterial milk community in different dairy livestock species [3,4,5]. The changes in the balance of the bacterial community and their mutualistic interactions with the host could have an impact on the animal’s health [6]. Hence, most studies on the milk microbiota of dairy ruminant species have focused on these changes and the differences between the microbial composition of milk samples obtained from healthy udders and those suffering mastitis or local inflammation [7,8]. These studies have allowed knowing the milk core microbiota for the different species, which refers to all taxa commonly found across all the samples analysed in each study. Recent studies have shown that the milk microbiome could be more complex than expected and that the results can be biased depending on the different analysis approaches applied, on the analysed milk fraction, and also on the sequencing platform used for its study [9,10]. In dairy cattle, the core microbiota has been shown to change when comparing different breeds [11]. Thus, the relationships between microbiota and udder health may not be applicable from one to other breeds within the same species.

In dairy sheep, a previous study of our research group presented the first characterisation of the milk core microbiota in this species by sequencing the 16S rRNA gene in milk samples from healthy ewes of the Spanish Assaf sheep [5]. Assaf is a highly specialised dairy breed integrated in Spain since 1977, which today has the highest census of dairy sheep population in the region of Castilla y León with a total of 126,841 ewes and 6322 rams (Data from: Ministerio de Agricultura, Pesca y Alimentación (Spain); https://www.mapa.gob.es/). This study described for the first time the sheep milk core microbiota and compared it with those reported in other species. In addition, this study characterised the microbiota of milk samples with different levels of somatic cell count (SCC), which is an indicator of the health status of the udder [5].

In the same geographical region of Castilla y León in Spain, Churra sheep are also exploited for milk production. Churra is a rustic, autochthonous breed from Castilla y León region with a milk selection scheme since 1986 [12]. The census of this breed has suffered a significant decrease in the last years due to the higher milk production level of foreign dairy specialised breeds (116,403 ewes and 2016 rams; https://www.mapa.gob.es/). In this context, the present study aims to delve into the characterisation of the milk microbiota of the autochthonous Spanish Churra sheep breed by exploiting 16S rRNA gene sequencing. Moreover, here, we have compared the microbiota composition pattern of the Churra milk samples analysed based on the different levels of somatic cell counts (SCC). The comparison of the 16S sequencing datasets generated here for Churra and in our previous study for Spanish Assaf has allowed us to assess the role of some factors that might influence the microbial composition of sheep milk samples.

## 2. Materials and Methods

### Churra Milk Sampling and Bioinformatic Data Analysis

In total, 212 milk samples from Churra ewes were included in this study. All the animals studied here did not show clinical signs of mastitis. The tested animals belonged to two different flocks (*n* = 145 and 67, were sampled for flock 1 and flock 2, respectively) from the region of Castilla y León (Spain), and each ewe was sampled once. Flock 1 had an intensive management system, whereas Flock 2 followed a semi-extensive management system, based on daily grazing. The sampling protocol used was the same as that described in our previous study on the Assaf breed milk microbiota [5]. Briefly, 100 mL of milk were collected into two 50 mL sterile containers; one sample was used for the DNA extraction and the other one for measuring SCC.

All the milk samples were transported to the laboratory under the same conditions using refrigeration and maintaining the samples at 4 °C. In order to generate comparable sequencing data with the previous study on the Assaf breed, all steps for DNA extraction were carried out in the same laboratory and over the same conditions than those described by Esteban-Blanco et al. [5]. The BiomeMakers^®^ (Valladolid, Spain) custom primers were used to amplify the hypervariable V4 region (Patent WO2017096385), and the Illumina Miseq platform (Illumina, San Diego, CA, USA) was used to perform the sequencing process. The raw data generated were analysed following the same bioinformatic pipeline described by Esteban-Blanco et al. [5]. Shortly, amplicon sequences variants (ASVs) were detected using the DADA2 pipeline [13], and the SILVA nr v.132 database [14] was used to perform the taxonomic assignment. Microbiota diversity of Churra milk samples was estimated with the Shannon index, which was calculated on the ASV rarefied data table. Following Gonzalez-Rodriguez et al. [15], a threshold to distinguish between healthy and subclinical mastitis ewes was set to 400,000 cells/mL. Hence, samples were distributed into two different groups based on SCC: “Healthy”, those samples showing SCC < 400,000 cells/mL and “SM” (subclinical mastitis) samples with SCC > 400,000 cells/mL. Basic statistics (average, standard deviation, range) of the SCC values observed for the two considered groups of milk samples and the result of the t-test contrast analysis performed for them are provided in Table S1. To explore bacterial diversity across Churra milk samples with different SCC levels, we followed the same approach to that of our previous study [5] and considered within the SM group two different groups based on the SCC observed levels: “SM1” group (400,000 < SCC < 2,000,000 cells/mL) and “SM2” (samples with SCC > 2,000,000 cells/mL), whereas the “H” group included those samples with less than 400,000 cells/mL.

An additional exploratory analysis was later performed to compare the microbiota composition of all the milk samples from the three flocks analysed by our research group, the two Churra flocks analysed in the present study and one Assaf flock previously analysed [5]. For that, we merged the taxonomic assignment tables generated for these two datasets, and DESeq2 [16] was used to identify the genera showing significant differences in relative abundance among the three flocks; these genera were filtered using an adjusted *p*-value cutoff of 0.05 and a log2fold change bigger than 1.5. The relationships of differentially abundant genera (DAG) among different combinations (Assaf vs. Churra-Flock1, Assaf vs. Churra-Flock2, and Churra-Flock1 vs. Churra-Flock2) were visualised using the VennDiagram R package (v1.6.20) [17].

## 3. Results

### 3.1. Microbiota and Diversity Profile in Churra Samples

The sequencing dataset of the hypervariable region V4 of the 16s rRNA gene included a total of 16.3 million raw reads for the 212 Churra milk samples under study. The length of the raw reads was 301 bp. After size filtering, reads with a minimum of 250 bp were kept for the next steps. The quality control and chimaera removal produced a total of 11.7 million quality reads with an average of 55,000 reads per sample. The overall number of ASVs detected by the DADA2 analysis for the 212 Churra samples was 2519; only 142 ASVs showed a relative abundance higher than 0.1% (81.8% of all of the analysed sequences).

**Figure 1:**
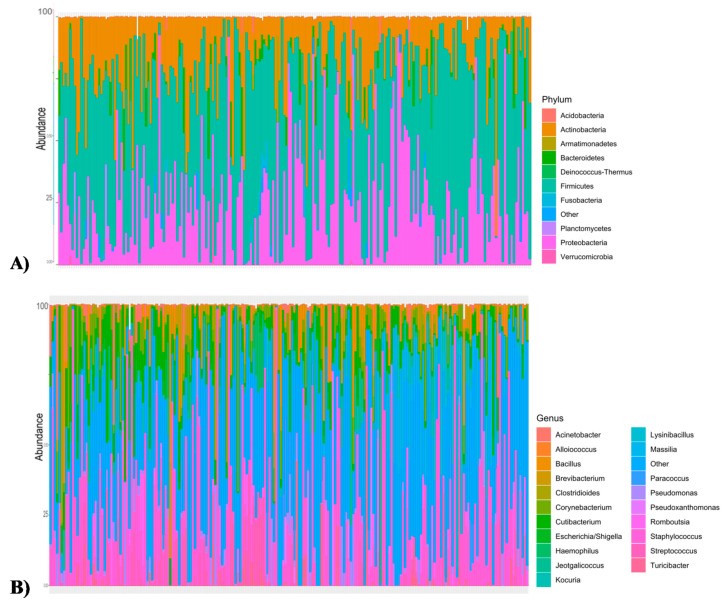
Barplot of taxonomic composition showing the relative abundance of the milk bacterial community in the Churra sheep breed at the phylum level (**A**) and at the genus level (**B**). Each bar represents a subject and each coloured box a bacterial taxon.

Once the ASV table was available, the performed taxonomy assignment identified a total of 31 phyla for the 212 Churra milk samples, and three and 28 were identified from the Archaea’s and Bacteria’s domain respectively. In the present work, the top most abundant Phyla that accounted for 97.4% of the abundance in the dataset were *Firmicutes* (50.28%), *Proteobacteria* (25.5%), *Actinobacteria* (18.9%) and *Bacteroidetes* (2.6%). However, minor phyla, each contributing less than the *Bacteroidetes* abundance and higher than 0.1%, accounted for the 2.2% of the total sequences; the most abundant minor phyla in this study were *Fusobacteria*, *Planctomycetes*, *Acidobacteria*, *Deinococcus*-*Thermus* (Figure 1A; Table 1). At the genus level, those taxa for which the bioinformatic analysis was unable to assign a specific genus were grouped under the “*Undefined*” label, and the “*Others*” label was also created to cluster genus with a proportion lower to 0.5%. The taxonomic assignment of data from the 212 Churra milk samples based on the 16S query database classified 16.9% and 5.9% of the taxa within the “*Others*” and “*Undefined*” labels, respectively. The predominant genera in Churra sheep milk were *Staphylococcus* (20.29%), *Cutibacterium* (6.27%), *Corynebacterium* (4.34%), *Streptococcus* (4.1%), *Massilia* (3.5%) and *Bacillus* (3.2%) (Figure 1B; Table 1). A comparison of these results with the results reported by our previous study for Assaf sheep milk samples [5] is provided in Table 1.

**Figure 2:**
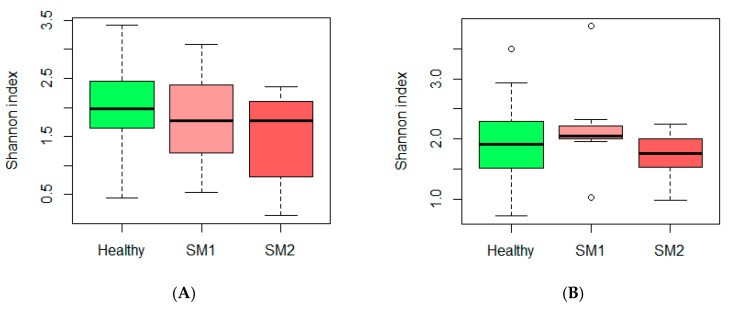
Boxplot graph representation of the alpha diversity Shannon index across groups of Churra milk samples defined based on somatic cell count (SCC) values. (**A**) Milk microbial diversity in Churra-Flock 1 (**A**) and Churra-Flock 2 (**B**) of Churra milk samples is represented for the three groups of samples defined in this work based on the SCC thresholds: “Healthy”, “SM1” (SCC > 400.000 cells/mL) and “SM2” (SCC > 2,000,000 cells/mL).

The core microbiota of Churra sheep milk described in this work, which is defined as the shared genera among all the Churra milk samples, included only two genera, *Staphylococcus* and *Escherichia/Shigella*. Considering the 10 top-most abundant genera present in the two groups of samples described based on SCC (“Healthy” and “SM”) and with at least a relative abundance of 1.5%, we observed that some genera present in the “Healthy” samples were not present in the SM groups (Table 2). Moreover, in the SM group, we observed a high increase in the relative abundance of *Staphylococcus* associated with a decrease in *Cutibacterium*, *Bacillus*, *Jeotgalicoccus*, and *Lysinibacillus*. Setting the sampling depth to 19,221, the estimated Shannon index values showed a slight reduction in microbial diversity among the three groups of milk samples defined based on the SCC value; however, no significant differences were observed among the three groups studied here. Moreover, the Shannon index found is around 2 for all Churra samples included in this study, regardless of the SCC value (Figure 2).

### 3.2. Comparative Study of the Milk Microbiota in Churra and Assaf Flocks

**Figure 3:**
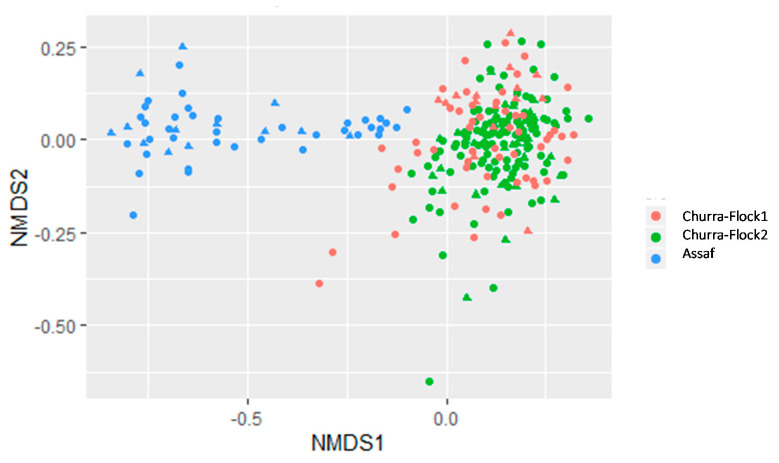
The similarity of bacterial communities between flocks. Non-metric Multidimensional Scaling (NMDS) ordination plot of the microbiota results obtained for sheep milk samples of Churra and Assaf sheep flocks. The distance between the samples is based on similarity in the amplicon sequences variant (ASV) composition of each sample calculated using the Bray-Curtis similarity index.

The joint analysis of Churra and Assaf milk samples identified a total of 15,692 ASVs and 1048 genera. Only 142 genera have a relative abundance higher than 0.1% and accounted for a total of 92%. The beta-diversity analysis performed for the Churra and Assaf datasets with the NMDS ordination analysis revealed a clear separation between the Assaf flock and the two Churra flocks. Although the Churra milk samples belonged to two different flocks, this analysis did not show apparent metagenome differences between the samples from different farms (Figure 3).

**Figure 4:**
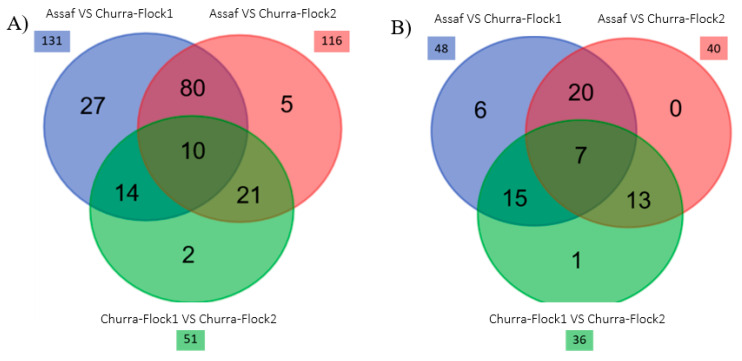
Differentially abundant genera (DAG) across the different flocks. (**A**) Venn diagram classifying all DAG reported by the DADA2 implemented pipeline. (**B**) Venn diagram classifying only DAG showing a relative abundance higher than 0.1%. Venn diagrams showing the relations between the DAG in the pairwise comparison of the considered subsets: Assaf versus Churra-Flock1 (blue), Assaf versus Churra-Flock2 (red), and Churra-Flock1 versus Churra-Flock2 (green). DAG were filtered with a threshold adjusted *p*-value ≤ 0.05 and an absolute log2 fold-change of ≥ 1.5. The total number of DAG for the different pairwise comparisons are indicated with an external square to the Venn diagram.

The whole dataset involving the microbiota results of the Churra and Assaf milk samples was subjected to a later analysis with the aim of identifying a potential breed-specific bacterial profile with DESeq2. The Venn diagram in Figure 4 shows that the overlap of differential abundance genera is more pronounced between breeds when the DESeq2 analyses included all ASVs than when the analysis included only the 142 most abundant genera. Overall, 159 DAGs were identified among the three flocks studied here, most of which were in the pairwise comparatives that include the Assaf milk samples (Figure 4A). Focusing on the most abundant genera (relative abundance > 0.1%) and by comparing the microbiota composition from Assaf, Churra-Flock1 and Churra-Flock2 milk samples, we identified seven genera shared among the three considered subsets. Only one of these seven common taxa was found among the 10 top-most abundant genera, *Massilia* (Table S2). The DESeq2 analysis revealed that its differential abundance is lower in the Assaf subset compared to Churra-Flock1 and Churra-Flock2 groups, while it is lower in Churra-Flock1 compared with Churra-Flock2.

The 20 common shared microbiota in the pairwise comparisons between the Assaf-VS-Churra-Flock1 and Assaf-VS-Churra-Flock2 might be considered as breed-specific genera. From those, we next examined only those genera that were present in the list of the 10 top-most abundant taxa (Table 2): *Staphylococcus*, *Cutibacterium*, *Lactobacillus*, and *Bacillus*. Details of the differential abundance results obtained with the DESeq2 analyses from these four genera are given in Table 3.

## 4. Discussion

For many years, the research work related to the bacterial composition in different tissues was based on the study of the contribution of single or few microorganisms [18]. The analysis of the microbial communities present in sheep’s milk has only been possible with the recent developments of massive parallel sequencing-based technologies, mainly through the analysis of the 16S rRNA gene.

To our knowledge, only one study previously reported by our research group has used this sequencing approach to characterise the sheep milk microbiota composition in a commercial population of the Assaf sheep breed [5]. In this study, we have applied the same methodology approach, analysing 16S rRNA V4 genes of bacteria, to characterise the milk microbiota in dairy ewes of the Spanish Churra breed. The DADA2 analysis performed identified 142 ASVs with relative abundance higher than 0.1% from a total of 2519 ASVs detected. This value is lower than that previously reported in Assaf milk samples by our group, which was 13,987 [5], although the entire biological diversity within samples was sufficiently captured (Figure S1). The accumulated abundance at the phylum level revealed that the more predominant phyla were *Firmicutes*, *Proteobacteria* and *Actinobacteria* (Table 1). These phyla have also been stated as prevalent taxa by other authors in different dairy livestock species such as dairy cattle, buffalo and sheep [3,4,5]. However, at the genus level, the total number of genera included in the core microbiota reported in this work for Churra sheep (*Staphylococcus* and *Escherichia/Shigella*) was lower than in the milk core microbiota described for other dairy species, such a cow, buffalo and goat, and that involves more than seven different genera [4,10,19,20]. The core microbiota reported here for Churra sheep also includes a lower number of genera than the five previously reported as the core microbiota of Assaf sheep milk: *Corynebacterium*, *Escherichia/Shigella*, *Lactobacillus*, *Staphylococcus* and *Streptococcus* [5]. When comparing the results of this work with the microbiota characterisation reported for Assaf milk (Esteban-Blanco et al. [5]), we assume that no batch effect is present between the two datasets (Churra and Assaf samples) because the transport, extraction and sequencing processes were performed following the same procedures. Hence, our characterisation of sheep milk microbiota in Assaf and Churra sheep breeds suggests that the milk microbiota is most diverse in Assaf than in Churra ewes, considering the two different flocks of Churra breed separately. In any case, this statement should be taken with caution as our experimental design does not allow us to separate the breed effect from the flock effect. Further studies would be needed to confirm this preliminary hypothesis.

Following the assessment presented in our previous work on the Assaf breed about the microbiota composition between samples with different SCC levels, we performed here a similar evaluation for Churra sheep, by dividing the 212 available Churra milk samples into the same groups previously defined according to the SCC levels, “Healthy” (165 samples with SCC < 400,000 cells/mL), “SM1” (33 samples with SCC > 400,000 cells/mL) and “SM2” (14 samples with SCC > 2,000,000 cells/mL). Interestingly, in contrast with the pronounced decrease observed in the microbiota diversity of Assaf milk samples with SCC > 4,000,000 cells/mL (Esteban-Blanco et al. 2019), Churra milk samples with extreme values of SCC did not show significant changes in the bacterial species richness (Figure 2A). These results may suggest that the breed factor not only influences the milk core microbiota but also how the SCC level influences the milk microbiota composition. Another potential explanation is that in those breeds for which milk microbiota diversity is lower, e.g., Churra, such composition is less influenced by changes in SCC levels. This might be related to the classic study of Dario and Bufano [21] where greater resistance to mastitis was demonstrated in less-productive breeds compared with more productive breeds. Supporting this, Gonzalo et al. [22,23] have reported slightly lower levels of SCC for two autochthonous dairy breeds, Spanish Churra and Castellana, compared with two other high milk productive breeds, Spanish Assaf and Awassi. In any case, to know if the different levels of basal microbiota diversity reported here between Assaf and Churra sheep breeds have any relationship with a potential higher resistance/susceptibility status to mastitis of any of these two breeds, further research including these two breeds under the same management will be needed.

Moreover, our additional analyses with the complete dataset, including Assaf and Churra information, suggested that the flock factor did not influence the milk microbiota of Churra milk samples. Although our experimental design is not appropriate to assess the effect of the breed factor, since it is confounded with the flock effect, the observations reported here would suggest that there is a clear distinction between the milk microbiota of Churra and Assaf milk samples exist. Moreover, the NMDS plot confirmed the higher microbiota diversity of Assaf milk samples, compared with Churra milk samples, as previously suggested by the Shannon index. However, to gain a global view of the influence of how the breed factor influences sheep milk microbiota, more studies are needed on this regard. Overall, the results presented here highlight the complexity of the sheep milk microbiome, as other authors have already stayed in relation to the study of the milk microbiome in dairy cattle [11]. The differences observed in the microbiota profiles between breeds could be related to the protective role of a balanced microbiota and resistance to infections. Further analyses based on the correlation between SCC and the microbial composition of milk from different sheep breeds should be carried out to explore the possible protection of specific microbiome patterns against pathogenic bacteria within the breed.

A differential pattern of microbial composition abundances between the two breeds studied here was obtained with the DESeq2 analysis. Shortly, considering genera with a value of fold change bigger than 1.5, we observed that *Cutibacterium, Bacillus*, and *Staphylococcus* were more abundant in Churra flocks; on the other hand, *Lactobacillus* was more abundant in the Assaf flock (Table 3). In Churra milk samples, the presence of *Cutibacterium* (formerly *Propionibacterium*), which is a common skin inhabitant [24] may suggest potential sample contamination. Moreover, no other studies in the field have reported the presence of this species in milk samples. On the other hand, Oikonomou et al. [7] claimed that geographical conditions and sampling sites have an impact on the microbiome detected in milk samples. These authors could differentiate samples from different flocks based on their microbial profile. However, in this work, the abundance of *Cutibacterium* is completely uniform across all 212 Churra samples collected across the two considered Churra flocks, which suggests that the sampling has minimised environmental contamination. Previous studies have shown that the *Bacillus* genus is frequently found in soil environments and on plants and is also able to spread during sub-optimal storage (8 °C) of pasteurised milk [25]. Few studies consider that *Bacillus* is part of the core microbiota of raw bovine milk [26], while other microbiota studies in dairy species that directly sampled from the teat of a cow, buffalo, sheep and goat did not identify this genus in the core microbiota [10]. Hence, the presence of *Bacillus* in Churra sheep milk will need to be confirmed by future studies. About *Staphylococcus*, it is one of the most cited dominant taxa in studies on milk microbiota in dairy species [4,20]. Moreover, these genera have been reported to show higher abundances in milk samples with mastitis or subclinical mastitis than samples without clinical signs of mastitis [4,10,27]. However, different *Staphylococcus* spp. appear to have different roles in relation to the development of this kind of infections [28]. In this study, we might relate the high abundance in *Staphylococcus* in Churra milk samples with the reported low microbial diversity value. Finally, regarding *Lactobacillus*, it is interesting to note that this genus has been recognised as a common microorganism in the core defined for healthy cow milk samples [20] and it is also reported as a genus that may inhibit mastitis pathogens [29]. Moreover, *Lactobacilli* are recognised as, for example, an important part of lactic acid bacteria (LAB) that are present in raw milk and traditional dairy products such as cheeses, yoghurt, and fermented milk [30], and it can contribute to cheese flavour and texture [31]. Future studies might identify *Lactobacillus* spp. to relate the differences of cheese making properties between Churra and Assaf and the higher proportion of this genus in milk from Assaf ewes.

## 5. Conclusions

By exploiting the massive parallel sequencing of the 16S rRNA gene, this study provides a first characterisation of the core microbiota of milk samples of Spanish Churra sheep, which involves genera such as *Staphylococcus* and *Escherichia/Shigella*. The evaluation of the microbiota pattern of milk samples with different SCC values suggested that in Churra sheep, there are some different genera between the two groups studied here (“Healthy” and “SM”). The milk microbiota from the two studied Churra flocks showed a much-limited diversity than that previously reported for a single flock of Spanish Assaf ewes, which is a highly specialised dairy sheep breed. It seems that both general microbial diversity and microbial taxonomy differ between different sheep breeds. Moreover, the exploratory study performed here reveals the presence of some breed-specific microbial genera in sheep milk from different breeds. Overall, the present work provides the first step into our understanding of the interactions between sheep milk microorganisms and factors such as breed and rearing system, paving the way to future studies aiming at assessing potential associations between the sheep milk microbiota and traits and issues of economic interest for the dairy industry (subclinical mastitis resistance, milk technological properties, milk traceability, and milk quality classification).

## Figures and Tables

**Table 1:** Relative microbial abundances for the top-most abundant phyla and genera identified through 16S rRNA gene sequencing in the milk samples of the two sheep breeds studied in this work, Churra and Assaf.

Level	Churra	Assaf (Esteban-Blanco et al. [5])
At phylum level	*Firmicutes* (50.28%) *Proteobacteria* (25.5%) *Actinobacteria* (18.9%) *Bacteroidetes* (2.6%)	*Firmicutes* (64.44%) *Actinobacteria* (14.25%) *Proteobacteria* (9.08%) *Acidobacteria* (2.7%) *Bacteroidetes* (2.3%)
At genus level	*Staphylococcus* (20.29%) *Cutibacterium* (6.27%) *Corynebacterium* (4.34%) *Streptococcus* (4.1%) *Massilia* (3.5%) *Bacillus* (3.2%)	*Staphylococcus* (16.8%) *Lactobacillus* (14.1%) *Corynebacterium* (8.8%) *Alloiococcus* (6.8%) *Streptococcus* (4%) *Romboutsia* (3%)

**Table 2:** Relative microbial abundances for the 10 top-most abundant genera identified by the microbiota analysis of the three groups of Churra milk samples defined based on SCC.

All Samples (*n* = 212)	Healthy (*n* = 166)	SM (*n* = 46)
*Staphylococcus* (20.3%)	*Staphylococcus* (14.1%)	*Staphylococcus* (42.6%)
*Cutibacterium* (6.3%)	*Cutibacterium* (7.1%)	*Cutibacterium* (3.4%)
*Corynebacterium* (4.3%)	*Corynebacterium* (4.3%)	*Corynebacterium* (4.5%)
*Streptococcus* (4.1%)	*Streptococcus* (4.3%)	*Streptococcus* (3.5%)
*Massilia* (3.5%)	*Massilia* (3.6%)	*Massilia* (3.1%)
*Bacillus* (3.2%)	*Bacillus* (3.7%)	*Enterococcus* (2.4%)
*Romboutsia* (2.6%)	*Romboutsia* (3.1%)	*Escherichia*/*Shigella* (2.1%)
*Pseudomonas* (2.4%)	*Pseudomonas* (2.6%)	*Mannheimia* (1.9%)
*Jeotgalicoccus* (2.2%)	*Jeotgalicoccus* (2.5%)	*Pseudomonas* (1.7%)
*Escherichia*/*Shigella* (2.2%)	*Lysinibacillus* (2.5%)	*Alloiococcus* (1.7%)

**Table 3:** Details of the differential abundance analysis for the four DAG commonly identified between the Assaf vs. Churra-Flock1 and Assaf vs. Flock2 contrasts and that were included in the list of 10 top-most abundant taxa (Table 2) (*p*-adj  <  0.05 | log2foldChange > 1.5).

Pairwiswe Comparations	Genus	Base Mean	log2FC	lfcSE	Stat	*p*-Value	*p*-Adj
Assaf vs. Churra-Flock1	*Cutibacterium*	5597.47	−11.55	0.43	−26.92	1.35 × 10^−159^	1.19 × 10^−157^
	*Bacillus*	1864.60	−4.96	0.62	−8.05	8.31 × 10^−16^	9.14 × 10^−15^
	*Staphylococcus*	27,203.72	−2.43	0.45	−5.45	5.17 × 10^−8^	3.03 × 10^−7^
	*Lactobacillus*	4126.47	5.85	1.14	5.15	2.65 × 10^−7^	1.23 × 10^−6^
Assaf vs. Churra-Flock2	*Cutibacterium*	5597.47	−10.07	0.48	−20.75	1.29 × 10^−95^	1.13 × 10^−93^
	*Bacillus*	1864.60	−4.65	0.70	−6.63	3.42 × 10^−11^	3.01 × 10^−10^
	*Staphylococcus*	27,203.72	−1.82	0.51	−3.58	3.48 × 10^−4^	1.13 × 10^−3^
	*Lactobacillus*	4126.47	4.15	1.29	3.20	1.35 × 10^−3^	4.25 × 10^−3^

## Supplementary Materials

The following are available online at https://www.mdpi.com/2076-2615/10/9/1463/s1, Figure S1: Rarefaction analysis of the assessment of ASV coverage. (A) Captured microbial diversity in Churra breed. (B) Captured microbial diversity in Assaf breed. Table S1: Basic statistics related to the somatic cell count (SCC) values observed in the milk samples analysed considering the two groups of milk samples defined here based on the threshold suggested by Gonzalez-Rodriguez et al. [15]: “*Healthy*” and “*SM*” (subclinical mastitis) samples. Table S2: Differentially abundant genera (DAG) reported with DESeq2 for milk samples analysed in the present work for three flocks of Spanish dairy sheep breeds.
