# Supplementary material for: The Milk Microbiota of the Spanish Churra Sheep Breed: New Insights into the Complexity of the Milk Microbiome of Dairy Species
Source: Animals (Basel). 2020 Aug 20;10(9):1463. doi: 10.3390/ani10091463 (PMC7552695; doi:10.3390/ani10091463)
Supplement: Supplementary file 1 [file animals-10-01463-s001.zip › Table_S1_animals-883282.pdf]

Table S1. Basic statistics related to the somatic cell count (SCC) values observed in the milk samples analyzed considering the two groups of milk samples defined here based on the threshold suggested by Gonzalez-Rodriguez et al. [14]: “Healthy” and “SM” (subclinical mastitis) samples

Group based on SCC*	Number of animals	SCC Arithmetic Mean	SCC SEM	SCC Geometric mean	SCC Geometric SD	SCC minimum value	SCC maximum value	T-test (p-value) between H and SM groups**
"Healthy"	166	115,668.7	5,373.4	99,001.6	1,745.1	24,000	377,000	p = 9.2E-24
"SM"	46	4,905,174.0	983,129.0	2,422,449.0	3,204.6	414,000	27,558,000	

* Two groups of milk samples were considered in this study based on the threshold suggested by Gonzalo et al. [14] : The "Healthy” group, including those samples showing SCC < 400,000 cells/ml and the “SM” (subclinical mastitis) group, which included samples with SCC > 400,000 cells/ml.

** Student t-test was performed to evaluate significant differences between the log-transformed SCC values of the two groups studied here.
