# Supplementary figures and images for: The Milk Microbiota of the Spanish Churra Sheep Breed: New Insights into the Complexity of the Milk Microbiome of Dairy Species
Source: Animals (Basel). 2020 Aug 20;10(9):1463. doi: 10.3390/ani10091463 (PMC7552695; doi:10.3390/ani10091463)

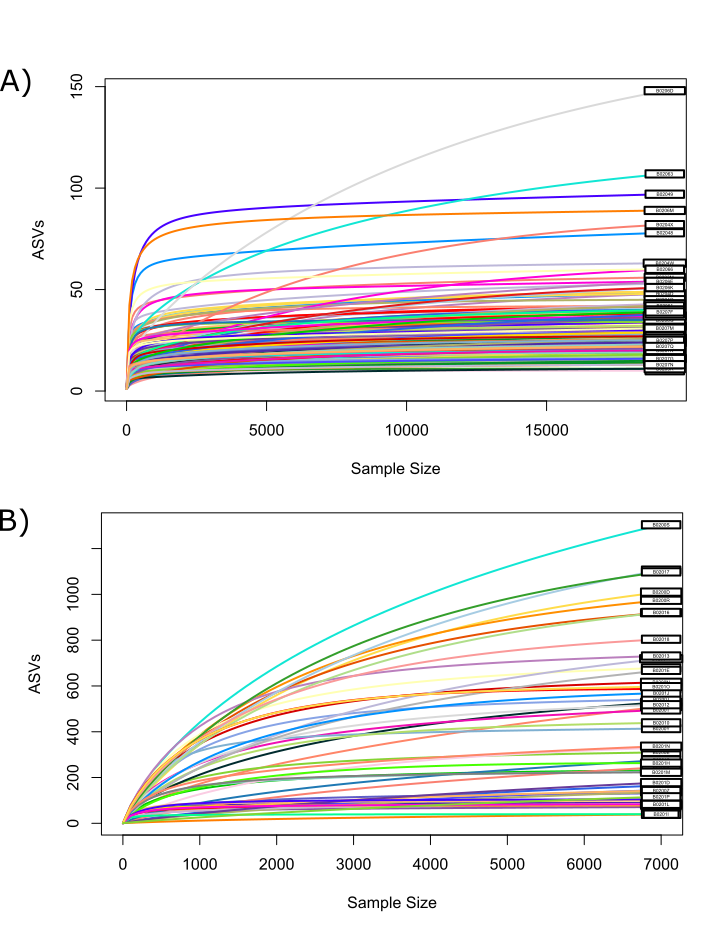

Supplement: Supplementary file 1 [file animals-10-01463-s001.zip › FigureS1.png]
